# Predictors for Moderate to Severe Acute Postoperative Pain after Cesarean Section

**DOI:** 10.1155/2016/5783817

**Published:** 2016-11-10

**Authors:** Natalia de Carvalho Borges, Lilian Varanda Pereira, Louise Amália de Moura, Thuany Cavalcante Silva, Charlise Fortunato Pedroso

**Affiliations:** Faculdade de Enfermagem, Universidade Federal de Goiás, Rua 227 Qd, 68, s/n, Setor Leste Universitário, Goiânia, GO, Brazil

## Abstract

*Background.* Moderate to severe postoperative pain affects performance of daily activities and it contributes to persistent postoperative pain. In patients submitted to cesarean section, this pain can also interfere with women's ability to care for their babies, to effectively breastfeed, and to satisfactorily interact with their children. Factors influencing the pain perception during the immediate postoperative period have not been widely pursued.* Objective.* To investigate the incidence and predicting factors of postoperative pain after cesarean section.* Methods.* A prospective longitudinal study with 1,062 women submitted to cesarean section. We collected sociodemographic, clinical, surgical, and health behavior data. We used the 11-point Numerical Pain and the Hospital Anxiety and Depression Scales. We performed logistic analysis to identify predictors of moderate to severe postoperative pain.* Results.* The incidence of moderate-severe postoperative pain was 78.4% (CI: 95%: 75.9%–80.8%). The preoperative anxiety (OR = 1.60; CI 95%: 1.22–2.30) and intrathecal morphine with fentanyl (OR = 0,23; CI 95%: 0.08–0.66) were significantly associated with moderate-severe postoperative pain report.* Conclusion.* The preoperative anxiety increases the risk of moderate-severe postoperative pain in women submitted to cesarean section. The intrathecal morphine with fentanyl added to bupivacaine was a protective factor against this pain.

## 1. Introduction 

Frequently, postoperative pain comes from lesion in tissues or organs generating stimulus perceived as painful [1]. When there is nerve lesion, stretching, or compression, neuropathic pain can be present [2].

This type of pain can cause a series of undesirable adverse events [3]. In addition, pain intensity equal to or higher than five (5) can bring losses for daily activities [4] and it is related to higher need of analgesics [5], thus, considered clinically unacceptable [6–8].

Intense acute postoperative pain has been an evident predictor for the persistence of this experience [9], because it can cause changes in plasticity of the nervous system [10] modifying pain perception [11]. In these cases, restoration of function is reduced if not impossible, and pain can be felt from nonnociceptive stimulus [12]. This can happen on cesarean section, a surgery that is frequently performed in women during fertile age [13].

Besides, pain felt by women submitted to cesarean section can harm their capacity to care for their babies, the first mother-child interactions, and the ability to effectively breastfeed [14].

Available studies provide evidence about some factors that can influence pain after cesarean section, as pain anticipation [15, 16], the need of medication [16], religion and spirituality [13], pain threshold [15, 17], and anxiety [16]; however, other factors should be investigated. The present study tries to contribute to the knowledge production on this theme and aims to determine the incidence and predicting factors of moderate to severe postoperative pain in women submitted to cesarean section.

## 2. Methods

### 2.1. Study Design and Local

The study is a part of a prospective open cohort, where recruitment of participants was during February of 2014 and July of 2015, in wards and apartments from a medium size private hospital, connected with the Unified Health System (SUS), in a city from the central region of Brazil (≈1,300,000 inhabitants in 2010). The hospital performs an average of 240 cesarean sections per month.

### 2.2. Participants

Women older than 14 years were admitted to a private hospital during immediate postoperative period after cesarean section. We excluded those in need of emergency cesarean section, with diagnostic of malignant disease, persistent hemodynamic instability, chronic use of opioids, with pain preventing participation, visual, hearing, or speech impairment, intraoperative intercurrence, and newborn death. One thousand sixty-two women participated and they gave written consent.

### 2.3. Data Acquisition

Nine trained interviewers performed interviews during pre- and immediate postoperative periods. Socioeconomic and demographic data (age, marital status, education, and socioeconomic classification); clinical condition (preoperative pain, active labor, anxiety, and depression), health behaviors (physical activity, alcohol consumption, and tobacco); surgical data (previous cesarean section, tubal sterilization concomitant with the cesarean section, and surgery duration); and intraoperative analgesia (intrathecal morphine and fentanyl plus IV and IM nonopioid analgesics) were collected during immediate preoperative period and in medical records. The assessment regarding presence of pain, intensity, and occurrence was done during the immediate postoperative period.

### 2.4. Instruments

#### 2.4.1. Brazilian Economic Classification Criteria

Brazilian Economic Classification Criteria was created by the Brazilian Association of Research Companies to classify individuals according to purchasing power. The instrument assesses the quantity of certain home appliances and bathrooms that families have at home and the educational level of the householder. These questions create a score varying from 0 to 46, in which individuals are classified as pertaining to classes “A1,” “A2,” “B1,” “B2,” “C1, C2,” “D,” and “E.” Class “A” refers to the highest class from the socioeconomic point of view and class “E” to the lowest. For this study, we pooled the classes, thus resulting in classes “A/B,” “C,” and “D/E.”

#### 2.4.2. Numerical Pain Scale

It was used to assess pain intensity. It is a unidimensional instrument that allows measurement of perceived pain intensity by numbers to quantify pain. This scale has 11 points (0 to 10), with point 0 (zero) representing no pain and point ten (10) the worst possible pain. The remaining numbers represent intermediate intensities of pain (1, 2, 3, and 4 = mild; 5 and 6 = moderate; 7, 8, 9, and 10 = severe) [18].

Clinically relevant postoperative pain was considered present when patients assessed it in their worse moment as intensity ≥ 5, that is, moderate to severe, and a cut-point was considered valid due to the increase in negative impact in physical and emotional dimensions of the individual [4, 19, 20].

#### 2.4.3. Hospital Anxiety and Depression Scale (HADS)

It is a useful instrument to assess changes in the emotional state of patients, as well as in the investigation of the presence or absence of clinically relevant titles of anxiety and depression. It is constituted by 14 items with four alternatives for answers to each one and seven questions referring to anxiety state and seven to depressive symptoms [21]. We used a translated and adapted version to Brazilian Portuguese [22].

### 2.5. Statistical Analysis

We presented continuous variables as mean and standard deviation (SD) and categorical variables as absolute and percentage values. We used Logistic Regression model for analysis of potential predicting factors for postoperative pain. The outcome used was the report of moderate to severe pain (intensity ≥ 5). The exposition variables included in the model presented a *p* value ≤ 0.10 in the univariate analysis. We assessed the magnitude of association by odds ratios with confidence intervals of 95%. The variables with *p* values < 0.05 were considered as significant predicting factors.

## 3. Results 

We counted 1122 women on the immediate cesarean preoperative period. Based on the study criteria, four (0.4%) were excluded due to newborn death, one (0.09%) due to hearing impairment, and eight (0.7%) because they reported intense pain during the preoperative period. From the 1109 women meeting inclusion criteria, 27 (2.4%) refused to participate in the study and 20 (1.8%) were discharged before data collection (1.8%), totalizing 1062 participants.

The sociodemographic, clinical, and surgical variables were showed in Table 1. All women received intrathecal bupivacaine 0,5% (mean = 12.3 mg; SD = 1.4) combined with morphine (mean = 86.5; SD = 12.2), in the intraoperative period. The intrathecal fentanyl was also administered to some of the patients (*n* = 35; mean = 21.5 mcg; SD = 3.3). Additional analgesia included simple analgesics, NSAIDs, and steroids. Approximately half of the subjects received intravenous dipyrone (45,6%) with an average dose of 1.740,3 mg (SD = 458.3). Intravenous dexamethasone 10 mg (6.6%) and intramuscular ketoprofen 100 mg (5,8%) were also used.

Most women reported severe pain after surgery that appears with higher frequency of movements (Table 2). The incidence of moderate to severe postoperative pain was 78.4% (CI 95% = 75.9%–80.8%).

We present potential predictors of moderate to severe postoperative pain after cesarean section on Table 3. In the multivariate model, patients that presented preoperative anxiety had increased risk of reporting postoperative pain as moderate to severe. The administration of fentanyl combined with morphine in the intraoperative period was a protective factor against moderate-severe pain report (Table 4).

## 4. Discussion

Our study found frequent postoperative pain in women submitted to cesarean section, of high intensity, despite advances in knowledge about the painful experience and drugs for its alleviation. Added to this, there is evidence of preoperative anxiety being a predictor for acute postoperative pain of high intensity and the administration of fentanyl combined with morphine in the intraoperative period being a protective factor against this pain.

Despite the relationship between anxiety and pain being targeted by researchers since the 1950s decade [23], we verified a scarce production of knowledge about this subject in women submitted to cesarean section.

The findings of this study corroborate with findings of Pan et al. [16], which evidence pointed anxiety, pain expectancy, and use of medications as predicting factors for postoperative pain.

At the beginning of investigations about the influence of anxiety in postoperative pain, a theory was proposed about the psychological stress (that can involve anxiety, fear, and other emotional answers), when facing a potentially threatening event, a surgery, for example, [23]. This theory, denominated as “the work of worry,” defended a curvilinear association between preoperative stress and patient recovery, in a way that extreme low or high anxiety levels during the preoperative period would cause elevated pain intensity during the postoperative period. Similarly, moderate anxiety levels would be associated with less pain intensity.

This moderate level of psychological stress would be the reflex to a self-preparation of the patient to experiment a situation associated with suffering. Thus, a patient with low levels of anxiety would represent an emotional lack of preparing to experience possible pain during the postoperative period, and patients with high levels of anxiety would have a higher predisposition for a higher awareness of the central nervous system [23, 24].

However, this theory was not confirmed in many posterior studies. Granot and Ferber [25] investigated the relationship between preoperative anxiety and pain intensity during the postoperative period after abdominal surgery and they found moderate anxiety levels constituting a risk factor for high levels of pain. These authors suggest that patients with low anxiety levels possibly predispose mechanisms to deal with their pain.

Also in a study conducted with 1000 women submitted to mastectomy, it was seen high levels of anxiety linked to the increase of experimental pain sensitivity and, also, to acute postoperative pain, contrary to Janis' theory [26].

The relationship between anxiety and postoperative pain has been studied in diverse surgical procedures. Studies show that preoperative anxiety significantly contributed to increase of pain intensity after dental surgery [27], mammoplasty [28], and total hip and knee arthroplasty [29].

Studies where anxiety is experimentally induced in humans also show the emotional state modulating pain, in a way that anxiety increases reactivity to pain, causing hyperalgesia [30–32].

Yet, the relationship between anxiety and pain is not always positive and unidirectional [33], as found by Gómez-de Diego et al. [34] in a study with 97 patients submitted to dental implant and by Kain et al. [35] in a double blinded clinical trial, placebo-controlled, with women submitted to hysterectomy, where a group received anxiolytic and the other not. In both studies, an association between these constructs was not seen.

The relationship found between anxiety and postoperative pain in women submitted to cesarean section is important because this symptom is highly prevalent in the period close to birth [36]. A study with 357 pregnant women found that 54.0% of women presented a high level of anxiety, at least in one of four antenatal assessments [37]. Besides, the thought of being totally conscious and immobile during the abdominal incision can generate anxiety [38]. Anxious women will be exposed to losses coming from high intensity pain during immediate postoperative and consequently during late postoperative period.

In this scenario, it becomes important to implement evidence-based strategies trying to reduce preoperative anxiety levels, for example, perioperative education and music therapy [39]. Perioperative education can be easily performed in this group of patients because routines of prenatal consultation favor this action. Such results contribute to the interaction quality between the binomial mother and child and the mother's capacity to conduct activities with the newborn.

The mother's wellbeing and her capacity to develop care activities to the newborn also depend on the quality of the analgesia obtained during the postoperative period [14]. The pain relief is a right of the woman and a need at the same time, once the nociception starts the release of catecholamines that harm the mother's body [40].

This study showed that fentanyl plus morphine and bupivacaine used in the spinal anesthesia were significantly associated with moderate to high postoperative pain. The patients who received those drugs via intrathecal route had less risk of reporting their pain as moderate-severe during the immediate postoperative period.

The spinal anesthesia has been widely used in patients undergoing cesarean section; however, bupivacaine alone does not provide extended analgesia in the postoperative period [41]. Intrathecal opioids are frequently used to achieve this analgesia [42]. The combination of fentanyl and morphine can provide a long-lasting analgesia with rapid onset [43].

Furthermore, fentanyl is the least intrathecal opioid to cause delayed respiratory depression [44], besides potentiating the intrathecal bupivacaine effect, which can reduce the doses of this drug and its side effects [45]. Research about intrathecal bupivacaine combined with other drugs has been conducted in order to evaluate the time of intraoperative anesthesia, postoperative analgesia, and the impact of this drug in the Apgar score. The results are promising although further researches are needed to corroborate these evidences.

Our study represents an advance in knowledge about postoperative pain in women submitted to cesarean section, allowing care planning for this population, despite some limitations that should be surpassed in future studies. One of them relates to the nonrandomized sample. Another limitation was the noninvestigation of variables related to pregnancy planning (if it was desired or not), a relevant question given the influence of biopsychosocial factors in pain perception.

The evidence opens space for deeper reflections about how the management in the surgical environment is between women submitted to cesarean section. Broader antenatal and perioperative assessments will allow intervening with higher chance to reach more satisfactory results that will meet the needs of pregnant and puerperal people in productive age who should have their health and wellbeing preserved.

## Figures and Tables

**Table 1:** Demographics and baselines characteristics.

Characteristics	Women (*n* = 1062) *n* (%)
Age, mean (SD)	25.1 (5.7)
Education ≥ 11 years^*∗*^	682 (64.4)
Marital partner	915 (86.2)
Socioeconomic class^†^	
A/B	348 (32.8)
C	628 (59.3)
D/E	84 (7.9)
Physically active^‡^	77 (7.3)
Alcohol consumption^‡^	73 (6.9)
Tobacco consumption^‡^	32 (3.0)
Active delivery	190 (17.9)
Previous cesarean section	369 (34.7)
Tubal sterilization^‡^	94 (8.9)
Surgery duration^§^	34.9 (10.8)
Preoperative pain	321 (30.2)
Preoperative anxiety^‖^	419 (40.2)
Preoperative depression^‖^	150 (14.4)
Intraoperative analgesics	
Intrathecal morphine plus IV and IM nonopioid analgesics	522 (49.2)
Intrathecal morphine	505 (47.6)
Intrathecal morphine and fentanyl plus IV and IM nonopioid analgesics	20 (1.9)
Intrathecal morphine and fentanyl	15 (1.4)

^*∗*^3 participants missing; ^†^2 participants missing; ^‡^1 participant missing; ^§^8 participants missing; ^‖^20 participants missing.

**Table 2:** Characteristics of preoperative pain in women after cesarean section.

	*n*	%
Pain at the surgical area		
Yes	984	92.7
No	78	7.3
Pain intensity^*∗*^		
Mild (1–4)	150	15.2
Moderate (5-6)	320	32.6
Severe (7–10)	513	52.2
When pain is felt		
Movement	729	74.1
Resting	15	1.5
Always	240	24.4

^*∗*^1 participant missing.

**Table 3:** Univariate analysis of potential predicting factors of moderate to severe postoperative pain.

	Postoperative pain					
	*n*	%	*β*	OR	CI (95%)	*p*
Age, mean (SD)	25.1 (5.7)		—	1.00	0.98–1.03	0.551
Education < 11 years	301	79.8	0.11	1.12	0.82–1.53	0.452
Without marital partner	120	81.6	0.23	1.25	0.80–1.96	0.311
Socioeconomic class						
C	493	78.5	−0,31	0,97	0.70–1,34	0.849
D/E	64	76.2	−0,16	0,85	0.48–1.49	0.571
Physically inactive	777	79.0	0.40	1.50	0.89–2.52	0.124
Alcohol consumption	59	80.8	0.15	1.17	0.64–2.14	0.605
Tobacco consumption	29	90.6	1.00	2.72	0.82–9.01	0.101
Active delivery	155	81.6	0.23	1.26	0.84–1.89	0.246
Previous cesarean section	281	76.2	−0.20	0.81	0.60–1.10	0.187
Tube sterilization	78	83.0	0.32	1.37	0.78–2.40	0.262
Surgery duration, mean (SD)	34.8 (10.8)		0.00	1.00	0.98–1.01	0.655
Preoperative pain	264	82.2	0.33	1.40	1.00–195	0.048
Preoperative anxiety	350	83.5	0.51	1.68	1.22–2.30	0.001
Depression	122	81.9	0.24	2.27	0.81–1.99	0.279
Intraoperative analgesics						
Intrathecal morphine plus IV and IM nonopioid analgesics	427	76.7	−0.22	0.80	0.59–1.07	0.140
Intrathecal morphine	406	80.4	0.22	1.24	0.93–1.67	0.140
Intrathecal morphine and fentanyl plus IV and IM nonopioid analgesics	16	80.0	0.09	1.10	0.36–3.32	0.864
Intrathecal morphine and fentanyl	7	46,7	−1.45	0.23	0.08–0.65	0.006

OR, odds ratio. CI, confidence interval.

**Table 4:** Multivariate analysis of moderate-severe postoperative pain predictors.

	*β*	OR_adjust_^*∗*^	CI (95%)	*p*
Preoperative pain	0.29	1.34	0.95–1.89	0.091
Preoperative anxiety	0.46	1.60	1.16–2.20	0.004
Intrathecal morphine and fentanyl	−1.44	0.23	0.08–0.66	0.006

^*∗*^OR, odds ratio adjusted by age. CI, confidence interval.
